# Inner Dialogical Communication and Pathological Personality Traits

**DOI:** 10.3389/fpsyg.2019.01663

**Published:** 2019-07-16

**Authors:** Małgorzata Łysiak

**Affiliations:** Department of Clinical Psychology, Institute of Psychology, John Paul II Catholic University of Lublin, Lublin, Poland

**Keywords:** inner speech, internal dialogues, self-talk, pathological personality traits, DSM-5

## Abstract

Dialogicality and its relation to personality traits have been extensively explored since the evolution of dialogical self theory. However, the latest edition of the Diagnostic and Statistical Manual of Mental Disorders (DSM-5) proposes a new hybrid personality disorder system and, thereby, a new model of pathological personality traits. As of now, there are no studies which show the relationships between self-talk, internal dialogicality, and pathological traits. Thus, the aim of this study was twofold: (a) to investigate the relationship between self-talk and pathological personality traits and (b) to explore the possible affinity between pathological structure of personality and dialogicality. A representative sample of 458 individuals from the non-clinical population, aged 18–67 (*M* = 30.99, *SD* = 10.27), including 52% women, completed three questionnaires: the Self-Talk Scale by Brinthaupt et al. (2009), the Internal Dialogical Activity Scale by Oleś (2009), and the Personality Inventory for DSM-5 by Krueger et al. (2012). To verify the correspondence between self-talk, internal dialogues, and pathological personality traits, the Pearson product–moment correlation coefficients (Pearson’s *r*) and canonical correlation analysis were used. The results supported the hypotheses about the specific relationship between internal dialogical activity and five crucial dysfunctional personality traits related to the hybrid DSM-5 system of diagnosis. People characterized as having emotional lability, anxiousness, and separation insecurity (high negative affectivity), with unusual beliefs and experiences, as well as eccentricity (high psychoticism), are prone to having ruminative and confronting dialogues. The correlation between pathological personality traits and self-talk were statistically significant, but the relationships are very small.

## Introduction

One of my patients in the session suddenly said: “Oh, my God, I’m talking to myself…do you think I’m abnormal?” When we started to question one of her dysfunctional beliefs, she started to go back to her past and analyze what she could have done if she had the baggage of experience she has today. Naturally, she had a dialogue-like conversation with herself. When she realized what she was doing, her reaction was as emotional as the first: “well, well, well! Not only do I talk to myself, but I am making a dialogue to myself. Are you sure I need this therapy? There is no need for explaining what happened next, but my patient’s observations led me to think about internal speech and internal dialogues as a special form of intrapersonal communication that requires more attention, especially research. Not without significance is the fact that I start by reflecting on a psychotherapeutic practice example, because the it shows how intrapersonal communication may work. Not only patients “talk to each other and conduct internal dialogue.” Such a process of intra-communication is a process studied by philosophers, literary scholars and psychologists. There are many types of inner speech, that fit into the category of intrapersonal communication, as well as individual differences in the frequency at which people experience internal speech (Hurlburt et al., 2013). Brinthaupt (2019) gives two hypotheses as an explanation of individual differences between people in terms of intrapersonal communication, which includes social isolation hypothesis and cognitive disruption hypothesis. In the context of pathological personality traits and intrapersonal communication the cognitive explanation is especially important. As we know from the cognitive-behavioral Beck’s theory the dysfunctional beliefs thought to underlie pathological behavior (Beck and Freeman, 1990). The counselor’s task in the conversation with the patient is to find these dysfunctional beliefs and help him/her to reformulate them. Dysfunctional beliefs are often expressed in the thoughts of patients, which often reflect their inner speech and inner dialogues. This is the first reason, why it is interesting to explore the nature of intrapersonal communication and whether it is related to personality traits. Beck and Freeman (1990) also claim “personality “traits” identified by adjectives such as “dependent,” “withdrawn,” “arrogant,” or “extraverted” may be conceptualized as the overt expression of these underlying (belief) structures” (p. 18). The intensity of the traits is different depending on the type of personality. Zawadzki et al. (1995) claim that narcissistic personality is associated with low agreeableness and high neuroticism, antisocial personality disorder with elevated neuroticism, low conscientiousness and agreeableness while obsessive-compulsive personality with elevated neuroticism and reduced openness to new experiences and compromise. According to the newest diseases classification DSM-5, the concept of personality traits and disorders is changed. The Diagnostic and Statistical Manual of Mental Disorders proposes a new hybrid personality disorders system, which entails a new model of personality traits.

Combining a number of individual differences in intrapersonal communication, clinical practice, Brinthaupt hypotheses and pathological personality traits, the purpose of the present study is to explore potential links between self-talk (e.g., Brinthaupt et al., 2009), internal dialogicality based on Hermans’ dialogical self theory (Hermans, 1996), and the construct of pathological personality traits based on the DSM-5 personality hybrid system.

Because people reflect on their inner experiences, we define inner speech as a dialogue with oneself which has a central role in self-regulation, self-reflection, and development (Alderson-Day and Fernyhough, 2015; Gut et al., 2018). Inner communication plays a crucial role in self-observation, where people can observe their experiences, emotions, and dispositions. It is considered to be the mental simulation of speech, as well as representative of cognitive function which is the main device for problem-solving, decision-making, and setting goals (Perrone-Bertolotti et al., 2014; Morin et al., 2018). Morin (2005, p. 5) suggests that “With inner speech one can engage in verbal conversations with oneself and replicate comments emitted by others (Cooley’s mechanism) or internalize others’ perspective (Mead’s mechanism).” With self-talk one can recall observations, emotions, appraisals made by others, and might imprint one’s own inner speech remarks on these recollections. Self-talk permits people to recreate the perspectives of others “in their private speech and to incorporate these multiple perspectives and into their concept of self” (DePape et al., 2006). Inner speech also allows people to regulate their mental states and can be involved in recalling past situations and emotions, also along with autobiographical memories (Morin, 2012). As with the past, internal speaking and internal dialogues are important in planning future situations and thinking, which can be helpful for creating psychological distance between the self and mental states created by the mind (Morin, 2005; Łysiak and Puchalska-Wasyl, 2018). Brinthaupt et al. (2009) identify the functions of self-talk which support the self-regulatory aspects of the self: social assessment, self-reinforcement, self-criticism, and self-management. The social assessment function refers to “self-talk related to a person’s social interactions” (Brinthaupt and Dove, 2012, p. 326). Focusing on positive events (e.g., feeling proud of something one has done or when something good has happened) is the self-reinforcement function, while regarding negative events (e.g., feeling discouraged about oneself or criticizing oneself for something one has said or done) refers to self-criticism (Brinthaupt and Dove, 2012). Lastly, self-management refers to giving oneself instructions or directions about what to do or say, or needing to figure out what to do or say, which is generally self-regulatory self-talk.

These functions express the dialogical nature of self-talk. Hermans’ dialogical self theory assumes the self “in terms of dynamic multiplicity of voiced positions in the landscape of mind intertwined as this mind is with minds of other people” (Hermans, 2003, p. 90). Relatively autonomous I-positions can interact with other I-positions, in open dialogical space and time, and reflect the different roles that a person can perform (e.g., values and ideals, identity, thoughts, and the ideas of others). I-positions, which are in constant motion, are associated with a probable story and they move from one self-position to another. The “conversations” between the positions give an expression of the experiences, beliefs, and feelings. A person can consider a problem from the point of view of the group to which they belong, express some aspect of themselves, feel important and separate in relation to other aspects of themselves, or they may represent a given person at different moments of their life (Łysiak and Puchalska-Wasyl, 2018). Internal dialogical activity seems to be very important in inner conflicts, where the positions confront different points of view. Self-dialogues may lead to re-evaluation of crucial individual experiences from different perspectives. Studies on the functions of internal dialogues concern support, substitution, exploration, bond, self-improvement, insight and self-guiding (Puchalska-Wasyl, 2007). The words “inner dialogues,” “inner speech” used in the field of psychology, have several hidden meanings, such as: *inner voice, verbal thinking, private speech, inner speaking, self-talk, internal monolog, internal dialogue* (e.g., Piaget, 1959; Vygotsky, 1962; Hermans, 2003; Brinthaupt et al., 2009). It is difficult to choose one appropriate definition, for the purpose of this research these words will be used interchangeably, with the main meaning being internal dialogical communication. However, it is important to distinguish the different dialogical activities. Self-talk is defined as self-directed speech, silent or loud, which mainly concerns self-regulatory functions (Brinthaupt, 2019), while internal dialogicality is an active process similar to interpersonal dialogues. Just as two people exchange views, thoughts, discuss or argue with each other, so two inner positions can interact with each other in similar ways. As there are many types of interpersonal communication, there are many types of intrapersonal dialogues, from identity dialogues to rumination or confrontation dialogues.

Although all positive and adaptive functions of internal speech are mentioned in the cited research, internal dialogues can also be non-adaptive and have negative consequences. First of all, inner speech has implications for patients in psychiatric conditions or with developmental disorders (Alderson-Day and Fernyhough, 2015). When inner speech becomes too intense, it can convert into pathological symptoms, such as insistent inner voices that characterize, for example, schizophrenia or redundant rumination, especially in social anxiety and depression (Perrone-Bertolotti et al., 2014). In psychotic disorders, inner speech is associated with auditory verbal hallucinations or hearing voices in the absence of the interlocutor. This is typical for a diagnosis of schizophrenia, but it is worth noting that there are no obstacles to this phenomenon appearing in the general population as well. Negative self-reflection – ruminations with negative thoughts – is one of the risk factors in affective disorders. Mainly cognitive-behavioral theories disclose data about maladaptive self-talk which is very important in developing anxiety and depression disorders (e.g., Padesky and Greenberger, 1995; Kendall and Choudhury, 2003). Calvete et al. (2005) explore how positive and negative content occurs in self-talk and how these, affect mood. The researchers used the Negative and Positive Self-Talk Scale and explored the connections for psychopathology traits. As expected, the trait “depression” was highly predicted by depressive self-talk and the trait “anxiety” by anxious and depressive self-talk. Positive-oriented self-talk has a connection with lower depression, but higher anger, while negative inner speaking correlates with anxiety, but not with depression (Alderson-Day and Fernyhough, 2015). Research by Brinthaupt et al. (2009) on frequency of self-talk showed that frequent self-talkers tended to be inwardly self-focused and had obsessive–compulsive tendencies. While the negative aspects of self-talk are correlated to anxiety, the positive ones appears in manic and narcissistic tendencies (Brinthaupt and Dove, 2012).

The conflict between various I-positions may cause neurotic problems if there are no efficient inner dialogue or assertive voices are suppressed by them (Stróżak, 2018). Puchalska-Wasyl and Oleś (2013) claims that doubtfulness is characteristic for providing dialogue. In some way the uncertainty is needed to provide the inner dialogue, while inner dialogues is one of the form for reducing the doubtfulness (Hermans and Hermans-Konopka, 2010). Research results by Chin et al. (2012) concerned uncertainty reveals that if people experience uncertainty, are likely to demonstrate those personality traits that may see as positive. Hermans and Hermans-Konopka (2010) postulate That living in times of uncertainty may contribute to engaging all form of dialogicality to reduce the doubtfulness and open the new ways of understanding the reality On the other hand the variety of possibilities, narrations, dynamic and constat changes may lead an individual to the most important value nowadays like being resilient and be ready to change.

The psychopathological side of living is linked to an American classification system, DSM (The Diagnostic and Statistical Manual of Mental Disorders); in the latest edition – DSM-5 – a new hybrid diagnostic system for personality disorders, with a dimensional pathological trait model, is proposed. It is also a five-factor trait model, but with a pathological version of the Five-Factor Model for normal personality (FFM); thus, it is called the “Pathological Big Five” (Krueger et al., 2011, 2012; Rowiński et al., 2018). In DSM-5, there are four criteria to diagnose personality disorder, but two of them are the most original: the level of personality functioning (Criterion A) and the model of maladaptive personality traits (Criterion B). The first one consists of self and interpersonal functioning and the second refers to personality traits (Waugh et al., 2017). The new DSM-5 model consists of 25 lower order personality facets that are classified into five higher order domains: negative affectivity, detachment, antagonism, disinhibition, and psychoticism. Negative affectivity (like FFM: neuroticism) involves tendencies to experience lability in feelings, especially unpleasant ones, the antagonistic or inactive behaviors. Detachment (like FFM: low extraversion) assessment of depressive feelings with anhedonia, general interpersonal withdrawal and suspiciousness. Antagonism (like FFM: low agreeableness) means callousness with tendency to manipulate and attention seeking. Disinhibition (like FFM: low conscientiousness) means irresponsibility, impulsivity, and risk-taking behaviors, with strict perfectionism. Psychoticism (like FFM: openness to experience) includes the features of eccentricity, odd and unusual beliefs and behaviors (Hopwood et al., 2012). In the FFM model there is an instrument to measure the traits; likewise, there is one in the DSM-5 model, where each trait is represented by a dimension scored using a dedicated instrument: namely, the Personality Inventory for DSM-5 (PID-5; Krueger et al., 2011). The analysis concerning the relationships between FFM and PID-5 confirmed four correlations, given that psychoticism and openness to experience are given no association (Góngora and Solano, 2017).

To date, several studies have been conducted to explore the nature and correlations of internal dialogical activity and personality, and they have not yielded the same results. Regarding personality traits (FFM), the studies confirmed that internal dialogical activity is moderately associated with openness to experience and neuroticism. On the basis of the research by Oleś et al. (2010), people with high neuroticism tend to conduct ruminative dialogues, whereas people with high openness have a tendency to use internal dialogues for identity clarification (Oleś and Puchalska-Wasyl, 2012). The same team researched attachment styles and internal dialogicality. It appears that secure attachment correlates positively with identity dialogues and negatively with ruminative dialogues, and anxious attachment correlates with the simulation of social relationships and ruminative dialogues. Avoidant attachment style has a negative relationship with supporting dialogues and identity dialogues, and a positive relationship with ruminative dialogues (Bątory et al., 2010; Oleś et al., 2010; Oleś and Puchalska-Wasyl, 2012). Studies on attachment styles and core beliefs, which are related to personality traits, considered that individuals with anxious style find others as difficult to understand with thoughts of having little control over outcomes in their lives, while people with secure attachment style are more assertive and interpersonally oriented (Platts et al., 2002). The research conducted by Zapała (2018) on imaginary dialogue and personality traits showed that openness to experience did not enhance the dialogical activity but was a predictor for creative dialogue as a personal dimension. Walasek’s (2018) on Eysenck’s personality types and internal dialogical activity confirmed the relationship between neuroticism and three types of dialogues: ruminative, confronting, and the simulation of social ones; no relationship between psychoticism and extraversion and inner communication was found. Her analysis also showed the connections between neuroticism and self-criticism, but only in a group of adolescents. While there are the correlations between internal dialogicality and FFM personality traits, Uttl et al. (2011) found very weak relationships between self-talk and big five personality traits. Given the frequency of self-talk, there is only a weak positive correlation with extraversion. An interesting study by Reichl et al. (2013) found negative correlations between loneliness and mental health, suggesting that people in weak or unsatisfactory relationships tend to use self-talk more frequent. Loneliness seems to be associated with uncertainty, and these two traits are very characteristic for personality disorders. This conclusion leads us to seek links between self-talk and pathological personality traits. As it was mentioned, uncertainty and doubtfulness are also linked to internal dialogicality, which also leads us to seek links between the inverted Big Five and internal communication.

In the outlined context, considering the adaptive and non-adaptive functions of inner communication and, to an extent, the DSM-5 hybrid personality pathological traits, the purpose of this research is to evaluate the degree to which pathological personality main domains influence variance in the functions of self-talk and types of dialogues. The main question of the study was posed: What are the relationships between the pathological personality traits and self-talk functions? What is the relationship between the pathological personality traits and internal dialogues? As this was an exploratory study, no hypotheses were formulated.

## Materials and Methods

The participants in the study were 498 individuals aged 18 to 67 (*M* = 30.99, *SD* = 10.27, 52% women). All of them completed three questionnaires: Self- Talk-Scale (STS), the Personality Inventory for DSM-5-SF (PID-5-SF) and Internal Dialogical Activity Scale (IDAS). The study was conducted by assistants, recruited from among psychology students. Each student invited 10 to 20 people from among their friends and acquaintances to take part in the study. All the participants were informed about the purpose of the study and signed their informed consent for participation. They filled questionnaires in the paper-pencil procedure and did not get any compensation. The study was conducted on a non-clinical sample, which means the results should be treated with caution. On the other hand, the dimensional approach presupposes the existence of specific traits that are found – with different degrees of intensity – in every person; a disorder is marked by a high intensity of these traits.

To examine the functions of the self-talk the Self-Talk Scale (STS) by Brinthaupt et al. (2009) was used. This self-report questionnaire includes sixteen items examines self-talk as described in relatively abstract terms and as generalized across time and situations. The participants responded to each item using a 5-point scale, in which 1 was “never,” 2 was “hardly ever,” 3 was “sometimes,” 4 was “fairly often,” and 5 was “very often.” The STS yields four scores for the scales including: social assessment, including wanting to replay something said to another person and imagining how other people respond to things one said (e.g., *I’m imagining how other people respond to things I’ve said*); self-reinforcement factor, which includes feeling proud of something when something good has happened (e.g., *I’m proud of something I’ve done*); self-criticism factor, which involves feeling discouraged about oneself and criticizing oneself for something said or done (e.g., *I should have done something differently*); and the self-management factor which entails giving oneself instructions or directions about what one should do or say, and needing to figure out what one should do or say (e.g., *I need to figure out what I should do or say*). The authors provide some initial evidence for the internal consistency, test–retest reliability, and construct validity of data collected from the measure (Brinthaupt and Dove, 2012). In the present study, the following alpha coefficients were obtained for the STS factors: social assessment, 0.76; self-reinforcement, 0.83; self-criticism, 0.75; and self-management, 0.73.

The Personality Inventory for DSM-5 (PID-5-SF) is a short form of a 220-item self-report inventory, PID-5, designed to assess the twenty-five pathological personality trait facets and the five higher-order domains of criterion B of the DSM-5 AMPD. The PID-5-SF-Adult is a 25-item self-rated personality trait assessment scale for adults aged 18 and older. It assesses five personality trait domains, including negative affectivity, detachment, antagonism, disinhibition, and psychoticism, with each trait domain consisting of five items. Each item on the measure is rated on a 4-point scale from 0–very false to 3–very true or often true. Each trait domain ranges in score from 0 to 15, with higher scores indicating greater dysfunction in the specific personality trait domain. Negative affectivity is defined as intense experiences of high levels of a wide range of negative emotions (e.g., anxiety, depression, guilt/shame, worry, and anger) and their behavioral manifestations (e.g., *I worry about almost everything*). Detachment is understood as avoidance of socioemotional experience, including both withdrawal from interpersonal interactions as well as restricted affective experience and expression, and, particularly, limited hedonic capacity (e.g., *I steer clear of romantic relationships*). Antagonism is a trait which puts the individual at odds with other people and includes an exaggerated sense of self-importance and a concomitant expectation of special treatment, as well as a callous antipathy toward others, encompassing both an unawareness of others’ needs and feelings, and a readiness to use others in the service of self-enhancement (e.g., *I crave attention*). Disinhibition is an orientation toward immediate gratification, leading to impulsive behavior driven by current thoughts, feelings, and external stimuli, without regard for past learning or consideration of future consequences (e.g., *People would describe me as reckless*). Psychoticism exhibits a wide range of culturally incongruent, odd, eccentric, or unusual behaviors and cognitions (e.g., *I have seen things that weren’t really there*), including both processes (e.g., perception, dissociation) and contents (e.g., beliefs). In the present study, the following alpha coefficients were obtained: negative affectivity, 0.74; detachment, 0.63; antagonism, 0.76; disinhibition, 0.76; and psychoticism, 0.70.

The Internal Dialogical Activity Scale (IDAS) by Oleś (2009) enables the assessment of the intensity of general dialogical activity in everyday life (general score) and seven kinds of internal dialogues measured by subscales: (1) pure dialogical activity (AD) – meaning spontaneous conduct of internal dialogues, thinking, and solving various issues in the form of dialogue (e.g., *I converse with myself*); (2) identity dialogues (ID) – internal dialogues aimed at better self-knowledge and answering identity questions, such as who am I, what is important to me, and what is the meaning of my life? (e.g., *Sometimes I debate with myself about who I really am*) (3) supportive dialogues (SD) – dialoguing which confirms beliefs, and supports or understands the imagined interlocutor, which may replace real conversations and give instructions (e.g., *In some stressful situations, I attempt to calm myself with my thoughts*); (4) ruminative dialogues (RD) – conducting internal dialogues about unpleasant topics, evoking difficult topics in thoughts, and pursuing them in the form of dialogue, accompanied by a sense of fatigue and frustration, and even a breakdown associated with internal dialogue activity (e.g., *After failures, I blame myself in my thoughts and discuss how the failures could have been avoided*); (5) confronting dialogues (CD) – conducting dialogues between two clearly separated parts of oneself, playing out internal conflicts in the form of dialogue (e.g., *Sometimes I think that my “good” side argues with my “bad” side*); (6) simulation of social dialogues (SS) – dialogues that are a continuation of conversations or a reflection of social dialogue relations: quarrels, discussions or exchanges of ideas (e.g., *Sometimes when I am preparing to talk to someone, I rehearse the conversation in my mind*); (7) taking a point of view (PV) – measures willingness to take a different viewpoint from one’s own, the viewpoint of another person, or to question one’s own opinion and attempt to assess events from a different personal perspective, and to objectify problems by looking at them from a new, different perspective (e.g., *Often in my thoughts I use the perspective of someone else*). Answers are given on a 5-degree Likert scale, ranging from 1–definitely disagree to 5–definitely agree. The higher the score, the higher is the intensity of internal dialogical activity.

In the present study, the following alpha coefficients were obtained for the IDAS subscales: pure dialogical activity, 0.78; identity dialogues, 0.82; supportive dialogues, 0.72; ruminative dialogues, 0.79; confronting dialogues, 0.80; simulation of social dialogues, 0.81; and taking a point of view, 0.65.

## Results

The basic statistics for each variable are given in Table 1. As this is a non-clinical sample, it is worth noting the kurtosis and skewness values for PID variables. The distribution is skewed to the left, but mostly in the levels of acceptance (sk < 1). Concerning the relationship between pathological personality traits and self-talk functions, a Pearson’s correlation was used. The analysis showed a significant but weak positive relationship between negative affectivity and self-criticism (*r* = 0.25), self-assessment (*r* = 0.15), and self-management (*r* = 0.14). Also, psychoticism and disinhibition are weakly correlated with self-assessment (*r* = 0.25 and 0.15), while self-management is correlated with psychoticism (*r* = 0.20) (Table 2).

**TABLE 1:** Descriptive statistics for self-talk, types of internal dialogues, and pathological personality traits.

	***M***	***SD***	**Min**	**Max**	**Range**	**Skew**	**Kurtosis**
SOCL_AS	10.56	3.67	4	20	16	0.03	–0.62
SELF-RE	11.12	3.73	4	20	16	–0.03	–0.69
SELF_CR	11.48	3.56	4	20	16	0.24	–0.43
SELF_ME	12.66	3.43	4	20	16	–0.19	–0.41
AD	18.27	5.08	6	30	24	–0.03	–0.60
ID	17.69	5.24	6	29	23	–0.17	–0.59
SD	20.28	5.08	7	34	27	–0.06	–0.08
RD	23.31	6.45	9	43	34	0.10	–0.25
CD	12.34	4.39	5	25	20	0.25	–0.67
SS	23.45	5.76	7	35	28	–0.40	–0.14
PV	15.72	4.20	6	30	24	0.08	0.11
NA	6.72	3.53	0	15	15	0.02	–0.67
DET	3.82	2.85	0	11	11	0.51	–0.65
ANT	2.64	2.73	0	12	12	1.19	0.97
DIS	4.08	3.22	0	15	15	0.66	–0.10
PSY	3.87	2.87	0	13	13	0.60	–0.26

*STS: SOCL_AS, social assessment; SELF_RE, self-reinforcement; SELF_CR, self-criticism; SELF_ME, self-management; IDAS: AD, pure dialogical activity; ID, identity dialogues; SD, supportive dialogues; RD, ruminative dialogues; CD, confronting dialogues; SS, simulation of social dialogues; PV, taking different points of view; PID: NA, negative affectivity; DET, detachment; ANT, antagonism; DIS, disinhibition; PSY, psychoticism.*

**TABLE 2:** Pearson’s correlation for pathological personality traits (PID) and self-talk functions (STS).

***Self-talk functions***				
**Personality traits**	**Social assessment**	**Self-reinforcement**	**Self-criticism**	**Self-management**
Negative affectivity	0.15^*^	0.08	0.25^*^	0.14^∗∗^
Detachment	0.04	–0.08	0.04	–0.01
Antagonism	0.12	0.10	0.06	0.04
Disinhibition	0.15^*^	0.08	0.12	0.11
Psychoticism	0.25^*^	0.12	0.12	0.20^*^

*p-*values were adjusted with Bonferroni’s correction. ^*^p < 0.01 and ^∗∗^*p* < 0.05.

To verify whether there was a relationship between the pathological personality traits and internal dialogues, the same statistical calculations were made. In the first step, Pearson’s correlation was used, which showed not very strong but positive relationships between personality domains and types of internal dialogicality (Table 3). The results showed that ruminative dialogues, confronting dialogues, and taking a different point of view are related to all pathological personality traits, while negative affectivity and psychoticism are related to all types of dialogues. Also, disinhibition is associated with more than half of the types of dialogues (Table 3). In view of these results, further analysis concerning a general exploratory question about the mutual relationship between the DSM-5 pathological personality traits and inner dialogues was carried out.

**TABLE 3:** Pearson’s correlation for pathological personality traits (PID) and internal dialogicality (IDAS).

***Types of internal dialogues***							
**Personality traits**	**AD**	**ID**	**SD**	**RD**	**CD**	**SS**	**PV**
Negative affectivity	0.24^*^	0.18^*^	0.21^*^	0.41^*^	0.29^*^	0.29^*^	0.22^*^
Detachment	0.02	0.05	0.08	0.26^*^	0.17^*^	0.09	0.14^∗∗^
Antagonism	0.13	0.06	0.09	0.19^*^	0.15^∗∗^	0.11	0.18^*^
Disinhibition	0.18^*^	0.11	0.18^*^	0.30^*^	0.27^*^	0.09	0.23^*^
Psychoticism	0.34^*^	0.32^*^	0.32^*^	0.41^*^	0.38^*^	0.24^*^	0.37^*^

AD, pure dialogical activity; ID, identity dialogues; SD, supportive dialogues; RD, ruminative dialogues; CD, confronting dialogues; SS, simulation of social dialogues; PV, taking a point of view. *p-*Values were adjusted with Bonferroni’s correction. ^*^p < 0.001 and ^∗∗^*p* < 0.01.

In order to answer the research question, canonical correlation analysis was used as a multivariate statistical model which allows the simultaneous prediction of multiple dependent variables from multiple independent variables. A canonical correlation analysis was conducted using the five personality traits as predictors and internal types of dialogue as criteria. The results of the correlation analysis refer to the direction of impact, nevertheless, such results should be treated with great caution.

The analysis provided three statistically significant functions (Table 4), but the second and the third explained only 8.8 and 4.7% of the remaining variance (unexplained by the first function). Therefore, only the first function, explaining 28.6% of the total shared variance between pathological personality traits (as predictors) and internal dialogical activity (as criteria), was considered in further analyses. As shown in Table 5, the first canonical variable representing DSM-5 personality traits is mainly loaded by psychoticism (to a high degree: 0.82), negative affectivity (high: 0.81) and disinhibition (moderate: 0.61). This canonical variable represents 41.7% of the variance shared by these three personality domains. The opposite canonical variable, created by inner dialogicality, represents 47.4% of the variance shared by all types of dialogues, and loaded mainly with ruminative dialogues (0.94), confronting dialogues (0.78), and taking different points of view (0.67).

**TABLE 4:** Canonical correlations with personality traits as predictors and internal dialogicality as criteria.

**Canonical function**	**Canonical correlation**	**Shared variance**	**Bartlett’s *Chi*^2^**	***p*-value**
1	0.54	28.6%	248.031	<0.001
2	0.30	8.8%	82.426	<0.001
3	0.22	4.7%	37.090	<0.01
4	0.13	1.6%	13.339	n.s.
5	0.10	1%	5.102	n.s.

*Predictors entered in the analysis: negative affectivity; detachment, antagonism, disinhibition psychoticism (PID), Criteria entered in the analysis: pure dialogical activity; identity dialogues; supportive dialogues; ruminative dialogues; confronting dialogues; simulation of social dialogues; taking a point of view (IDAS). n.s. - nonsiginificant.*

**TABLE 5:** Results for the first canonical function.

	**Loadings**	**Loadings squared**	**Percent of variance of the set variables explained by:**	
			**their own canonical variable**	**the opposite canonical variable**
*Predictor set*			41.7%	11.9%
Negative affectivity	0.81	0.67		
Detachment	0.45	0.20		
Antagonism	0.41	0.17		
Disinhibition	0.61	0.37		
Psychoticism	0.82	0.68		
*Criterion set*			47.4%	13.5%
Pure dialogical activity	0.64	0.41		
Identity dialogues	0.54	0.30		
Supportive dialogues	0.60	0.36		
Ruminative dialogues	0.94	0.87		
Confronting dialogues	0.78	0.60		
Simulation of social dialogues	0.57	0.33		
Taking different points of view	0.67	0.45		

Pathological personality traits and inner dialogicality have much in common; 28.6% of shared variance is quite substantial. The redundancy analysis shows that the latent variable, personality traits, explains 13.5% of internal dialogicality variability, whereas the particular types of dialogue explains 11.9% of DSM-5 personality traits.

Because canonical loadings with the same sign indicate a positive correlation of the variables, it could be said that the higher negative affectivity, psychoticism, and disinhibition, the higher is the degree of ruminative dialogues, confronting dialogues, and taking different points of view. Thus, those with a greater intensity of emotional lability, anxiousness, and separation insecurity (high negative affectivity) with unusual beliefs and experiences, as well as eccentricity (high psychoticism) and a tendency to be irresponsible and impulsive (high disinhibition), are prone to present ruminative, confronting dialogues, as well as taking different points of view.

To establish whether there were correlations among self-talk functions and internal dialogical activity, r-Pearson’s correlations among the STS and IDAS subscales were performed. The results are presented in Table 6. All correlation coefficients are positive and significant and are within the limits 0.20–0.46. The highest, but still moderate correlations are between pure dialogical activity and social assessment (0.46). Social assessment correlates with simulation of social dialogues (0.45). Pure dialogical activity and supportive dialogues have moderate correlations (around 0.4) with self-criticism and self-management.

**TABLE 6:** Correlations among subscales of types of internal dialogues (IDAS) and self-talk (STS).

	**SOCL_AS**	**SELF-RE**	**SELF_CR**	**SELF_ME**
AD	0.46^*^	0.33	0.44^*^	0.45^*^
ID	0.33	0.33	0.37	0.38
SD	0.40^*^	0.34	0.40^*^	0.41^*^
RD	0.26	0.20	0.34	0.21
CD	0.26	0.26	0.33	0.23
SS	0.45^*^	0.25	0.38	0.40^*^
PV	0.39	0.32	0.32	0.31

*p-*values were adjusted with Bonferroni’s correction ^*^p < 0.05. STS: SOCL_AS, social assessment; SELF_RE, self-reinforcement; SELF_CR, self-criticism, SELF_ME, self management; IDAS: AD, pure dialogical activity; ID, identity dialogues; SD, supportive dialogues; RD, ruminative dialogues; CD, confronting dialogues; SS, simulation of social dialogues; PV, taking different points of view.

## Discussion

The purpose of this research was to investigate the relationship between inner speech and pathological traits, using the model described in DSM-5. Two specific objectives were set: (1) analyze the relationships between the functions of self-talk by Brinthaupt et al. (2009) and DSM-5 pathological personality traits; (2) explore the possible affinity between pathological structure of personality and types of internal dialogical activity. Two overall findings were observed. With regard to the results, the lack of any significant correlation between functions of self-talk and DSM-5 pathological traits are noteworthy, while there is correspondence between inner dialogicality and personality pathological domains. The results of the canonical correlation showed quite substantial correspondence between DSM-5 personality traits and the types of internal dialogicality. A common variance exceeds nearly 30% and shows a clear affinity between the two sets of variables.

In light of the obtained results, the weak correlations between self-talk and pathological personality traits and, at the same time, the relationship between the pathological big five and types of internal dialogicality are puzzling. The first explanation of these results is related to the relationships between the internal speech objects. It is worth noting that the correlations between functions of self-talk and internal dialogical activity are not very strong. The strongest ones are between pure dialogical activity, supportive dialogues, and simulation of social dialogues with social assessment, self-criticism, and self-management as functions of self-talk. It is worth noting that although the strength of correlations is moderate, it does not mean that it is invalid. According to analysis of the definitions of these (functions and types) and research (e.g., Padesky and Greenberger, 1995; Calvete et al., 2005), it can be assumed that inner speech might be positive and negative. When combined with internal dialogical activity, functions of self-talk seem to be much more correlated with positive internal speech. A special relationship exists between self-criticism and supportive dialogues, which may suggest that critical self-reflection and dialoguing is a part of “productive” life (Hermans and Hermans-Konopka, 2010, p. 123). Confronting negative events might also be positive, especially in the process of self-reflection, when people retreat to their negative experiences or feelings to positively trigger an internal dialogue, which is supportive. McCarthy-Jones and Fernyhough (2011) distinguished four types of inner speech: *dialogic inner speech* – backward and forward conversational quality; *condensed inner speech* – a short, fragmentary form of inner speech; *other people in inner speech* – a representation of others’ voices or what someone else would say; and *evaluative/motivational inner speech* – which means judging or assessing one’s own behavior. The results indicate that evaluative/motivational inner speech and dialogic inner speech were most commonly chosen. Such a situation may relate in particular to the role of the critical internal voice, which in a healthy person can play a constructive mobilizing role. However, this interpretation should be treated with caution, as the study concerned the intensification of pathological features, hence the critic rather intensified his disadaptive strategies. According to this research, it is likely that the scales of internal dialogical activity and self-talk complement each other but explore different aspects of internal speech. These results may support that self-talk functions are not associated with pathological personality traits, although Brinthaupt et al. (2009) showed that self-talkers have obsessive–compulsive tendencies and tended to be inwardly self-focused.

While there is no correlation between functions of self-talk and personality pathological traits, canonical correlation analysis revealed a main pattern which is reflected in negative affectivity and psychoticism as predictors, and ruminative and confronting dialogues as criteria. With higher emotional lability, anxiousness, submissiveness, insecurity (negative affectivity), higher unusual beliefs and experiences, and eccentricity (psychoticism), people are prone to having dialogues which are focused on unpleasant themes, usually conducted with frustration (ruminative dialogues) and dialogues where two strictly divided parts of oneself tries to resolve internal conflict (confronting dialogues). In this context, the obtained results may be seen as DSM-5 pathological big five as reversed Five-Factor Model. Studies on dialogicality and FFM show that internal dialogical activity is associated with openness to experience. There are also low but significant correlations between dialogical activity and neuroticism and, more interestingly, only with two types of dialogue: ruminative and confronting (Puchalska-Wasyl et al., 2008; Oleś et al., 2010). The results from the research on the DSM-5 pathological personality and FFM personality models confirm a strong correlation between general traits and pathological traits (Krueger et al., 2012; Quilty et al., 2013; Thomas et al., 2013; Strus et al., 2017): negative affectivity with neuroticism (positive), detachment with extraversion (negative), antagonism with agreeableness (negative), and disinhibition with conscientiousness (negative). Ambiguous results were obtained by comparing psychoticism and openness with experience – some research found a relationship between these two traits (e.g., Thomas et al., 2013; Chmielewski et al., 2014), while others did not find any association (Quilty et al., 2013; Suzuki et al., 2015). Due to the potential for integrating models of normal and abnormal personality, the results on dialogicality appear to be compatible because negative affectivity is the counterpart of neuroticism and psychoticism is a counterpart of openness to experience. In both models of personality traits, ruminative and confronting types of dialogue are most characteristic. This would suggest the dimensional approach presupposes the existence of specific traits that are found with different degrees of intensity in every person and a disorder is marked by a high intensity of these traits. Personality traits (normal or pathological) participate in explaining the inner communication in its adaptive and non-adaptive functions and types.

Rumination in the categories of inner speech and dialogicality is the aspect of negatively experienced positions which dominate the self. When “ruminating,” *I* is constrained by the cluster of internal and external positions that are accessible, but they do not allow any exit. It is like prison from which a person cannot escape. A lack of any innovation and the dominance of one position is the reason why ruminating seems to be more monological than dialogical (Hermans and Hermans-Konopka, 2010, p.176). Positive inner speech is not accessible while ruminating. It seems compatible with these domains of pathological traits – negative affectivity and psychoticism – where high levels of a wide range of negative emotions, such as anxiety, depression, or worrying, and a wide range of eccentric, bizarre behavior, appearances, and/or speech, and having strange and unpredictable thoughts, may intensify the ruminative dialogues. It is like a “vicious cycle” such as in experiences of, for example, anxiety disorders: If a person has most of the features within the domains of negative affectivity or psychoticism, it makes sense that she or he can try do things that are opposite to the features. The more the person tries to not dialogue in a negative way, the more difficult it is. Although it may be possible for a non-clinical population, it may be very difficult for people with personality disorders.

Confronting dialogues define dialogues where two internal voices are in conflict and they try to push an individual in different, sometimes opposite, directions. This “war” in the mind can cause a lot of consequences in feelings or behavior. It may bring tension or frustration, yet can be developmental for the self, even leading to creative insight. Intensity and frequency of internal confronting dialogues causes emotional exhaustion and may become maladaptive. The relationship between psychoticism and negative affectivity and confronting dialogues confirms that if the personality develops into disorder, the loading of confronting dialogues is stronger. According to Morin (1993) the discrepancies between perspectives are accompanied by negative emotionality and self-awareness is constricted. At first sight, confronting dialogues usually seem to be accompanied by negative emotions, which cause discomfort and inconvenience; however, they are stronger if loaded by the traits where dissociative experiences and feelings of nervousness are present. Referring to cognitive behavioral literature (Beck and Freeman, 1990; Padesky and Greenberger, 1995), negative thoughts and negative internal speaking can cause non-realistic beliefs, which are related to personality traits. Negative thinking is supposed to be balanced by positive thoughts, which a person can integrate into their overall generally positive and emotionally healthy sense of self (e.g., Padesky and Greenberger, 1995). The obtained results are worth looking at in terms of clinical implications. As it has already been mentioned, the research on intrapersonal communication is a trend in practice toward integrating cognitive behavioral insights, where clinical psychologists, psychotherapists often try to change the content of inner speaking to help their clients alter emotional responding and function in more adaptive way. Imaginable dialogues stimulate thinking as much as the real one and it is more effective in constructing the solution (Staudinger and Baltes, 1996). Cognitive-behavioral interventions, especially while using “experimental techniques” to explore the dialogues, can be easier and quicker to use as we know the pathological traits as predictors and the types of dialogues as criteria (e.g., classic empty chair, dialogical temporal chair technique; Łysiak, 2017). These techniques can be useful to reconstruct ruminative or confronting dialogues in more effective way. Furthermore, if the pathological personality structure is known for psychologist, it will be useful to check the intensity of internal dialogical activity to plan different kind of interventions for example to change the emotions these dialogues may cause. This last remark relates to a question for further research: Can dialogical activity foster overcoming problems related to personality disorders and, if so, under which conditions and using which kind(s) of dialogical activity?

This study has some limitations. First, it is a strictly correlational study based on self-report questionnaires. Second, the sample consisted only of adults from one country and, although representing different kinds of academic educations or coming from different areas of the country, the research should be replicated in a different population. The study was conducted on a non-clinical sample; even if a disorder is marked by a high intensity of pathological traits, the clinical sample should be examined in further research to increase confidence in the results. The next limitation of the study is the procedure of participants recruitment. The researcher’s assistants were psychology students, who had the guideline about the sample specification and the criteria of data collection. But there is a possibility that they engaged their friends and acquaintances. This is also the reason to treat the results with caution. Another limitation is that the results regarding the relationships with the DSM-5 pathological personality traits were limited to basic personality traits only. The current study checked only the main domains of traits and their connections to inner speech, but an investigation of the lower facets is an important issue and might provide more information. A better understanding of the relationship between the DSM-5 personality trait structure and inner dialogicality needs further exploration.

In summary, the findings from this research concerning the relationships between pathological personality traits and inner communication allow us to identify a main role of two out of five pathological personality traits which mainly favor two out of seven types of internal dialogicality.

## Data Availability

All datasets generated for this study are included in the manuscript and/or the supplementary files.

## Ethics Statement

This study was carried out in accordance with the recommendations of “name of guidelines, name of committee” with written informed consent from all subjects. All subjects gave written informed consent in accordance with the Declaration of Helsinki. The protocol was approved by the Committee on Ethics in Scientific Research of the Institute of Psychology in John Paul II Catholic University of Lublin.

## Author Contributions

MŁ prepared the manuscript with the help of students who collected the data.

## Conflict of Interest Statement

The author declares that the research was conducted in the absence of any commercial or financial relationships that could be construed as a potential conflict of interest.
